# Outcomes of surgical treatment in patients with anorectal fistula cancer

**DOI:** 10.1186/s40792-021-01118-6

**Published:** 2021-01-26

**Authors:** Katsuya Osone, Hiroomi Ogawa, Chika Katayama, Yuta Shibasaki, Kunihiko Suga, Chika Komine, Naoya Ozawa, Takuhisa Okada, Takuya Shiraishi, Ryuji Katoh, Makoto Sakai, Akihiko Sano, Takehiko Yokobori, Nozomi Matsumura, Makoto Sohda, Ken Shirabe, Hiroshi Saeki

**Affiliations:** 1 Department of General Surgical Science, Graduate School of Medicine, Gunma University, 3-39-22 Showa-machi, Maebashi, 371-8511 Japan; 2 Division of Integrated Oncology Research, Gunma University Initiative for Advanced Research (GIAR), Maebashi, Gunma Japan; 3 Department of Human Pathology, Gunma University Graduate School of Medicine, Maebashi, Gunma Japan

**Keywords:** Anorectal fistula, Mucinous adenocarcinoma, Hyperthermo-chemoradiotherapy, FDG-PET/CT, Crohn’s disease

## Abstract

**Background:**

No standard treatment for anorectal fistula cancer, such as multidisciplinary therapy, has been established due to the rarity of the disease. Herein, we investigated patients with cancer associated with anorectal fistula who underwent surgery to clarify the clinicopathological characteristics and to propose future perspectives for treatment strategies.

**Case presentation:**

Seven patients with cancer associated with anorectal fistula who underwent rectal amputation in our institute were analyzed with regard to clinical characteristics, pathological findings, surgical results, and prognosis. Four cases had Crohn's disease as an underlying cause. All seven cases were diagnosed as advanced stage. Preoperative [^18^F]-fluoro-2-deoxy-d-glucose (FDG)-positron emission tomography/computed tomography (FDG-PET/CT) showed abnormal FDG accumulation in six cases including four mucinous adenocarcinomas. Three cases that received preoperative hyperthermo-chemoradiotherapy achieved pathological R0 resection. Postoperative recurrence was observed in four cases including three with Crohn's disease and one resulting in death.

**Conclusions:**

Anorectal fistula cancer is rare and difficult to be diagnosed at early stages. Mucinous adenocarcinoma associated with anorectal fistula tends to exhibit abnormal FDG accumulation by FDG-PET/CT unlike common colorectal mucinous adenocarcinoma. Preoperative hyperthermo-chemoradiotherapy may be effective in obtaining pathological complete resection.

## Background

Anorectal fistula cancer is a rare disease. There are a few case reports concerning this disease [1, 2], while no clinical studies based on a large enough cohort exist. There are many unclear points regarding its pathophysiology and clinical features. The origin of the cancer is reported to be the fistula tract or the anal gland [3]. One of the main clinical problems in anorectal fistula cancer is the difficulty of early diagnosis [4]. Additionally, no standard treatment for the disease, such as multidisciplinary therapy including surgery, has been established yet. Since these issues may be caused by the rarity of the disease, the accumulation of evidence obtained from cases that received treatment in real-world practice is thought to be valuable.

In this study, we summarized data from seven patients with anorectal fistula cancer who underwent surgery at our institute. We used preoperative therapy, such as hyperthermo-chemoradiotherapy (HCRT) [5–7], followed by surgery for some of these cases. The purpose of this study is to clarify the clinicopathological characteristics and surgical results and to propose future perspectives for treatment strategies for anorectal fistula cancer.

## Case presentation

### Patients

Seven patients with anorectal fistula cancer who underwent surgical resection in Gunma University Hospital between 2008 and 2018 were included in this retrospective study. Hospital patient records were reviewed for tumor characteristics and patient outcomes. The tumor stage and disease grade were classified according to the 8th edition of the TNM classification of the International Union Against Cancer (UICC) [8]. This study was approved by the Institutional Review Board for Clinical Research at the Gunma University Hospital (Maebashi, Gunma, Japan) (The approval number is HS2019-140.) and have, therefore, been performed in accordance with the ethical standards laid down in the 1964 Declaration of Helsinki and its later amendments. Patient consent was obtained via the opt-out method.

### Preoperative hyperthermo-chemoradiotherapy (HCRT)

The preoperative HCRT was performed as described in the previous papers [5–7]. In brief, radiation treatment was delivered by 10-MV X-rays through a three-field box technique. The clinical target volume encompassed the primary tumor and the entire mesorectal tissue. The total radiation dose was 50 Gy, with daily fractions of 2.0 Gy on five consecutive days per week. Chemotherapy consisted of capecitabine (1700 mg/m^2^ per day) given 5 days a week for 5 weeks on the day of radiation. Five hyperthermia sessions were performed once a week with 8-MHz radiofrequency capacitive heating equipment (Thermotron-RF 8; Yamamoto Vinita Co., Ltd., Japan).

### Surgical treatment

Rectal resection was performed using the principle of total mesenteric resection [9], which preserved the pelvic autonomic nerves, and abdominoperineal rectal amputation was performed in all cases. Patients who received preoperative HCRT underwent surgery approximately 8 weeks after its completion. Evaluation of postoperative complications was performed by the Clavien–Dindo classification [10]. Postoperative follow-up of all patients was done every 3 months. CT of the abdomen and thorax was performed every 6 months.

### Preoperative patient characteristics

Table 1 shows preoperative patient characteristics. The median age of cases was 65 years, with a male-to-female ratio of 5:2. Most cases were diagnosed by the examination or operation of anal fistula, and the diagnosis was performed by endoscopy or biopsy under local anesthesia. Pretherapeutic clinical stage was IIA in one case, IIB in three, and IIIB in three. Preoperative CEA was positive in three cases and [^18^F]-fluoro-2-deoxy-d-glucose (FDG)-positron emission tomography/computed tomography (PET/CT) showed abnormal FDG accumulation in all six cases examined. Preoperative HCRT was performed in three cases with suspected invasion into other organs, and four cases had Crohn's disease. The cause of the anal fistula in patients without Crohn's disease was probably an anal gland infection.

**Table 1:** Preoperative characteristics of the seven patients with cancer associated with anal fistula

Case	Age (year)	Gender	Diagnostic opportunities	Crohn’s disease and duration of Crohn's disease (year)	Duration of anal fistula (year)	Preoperative therapy	Pretherapy cStage	CEA positive	Biopsy methods	PET positive
1	47	Female	Anal stenosis	(+), 23	No date	HCRT	IIIB	(-)	General anesthesia	( +)
2	41	Male	Routine surveillance	(+), 27	No date	HCRT	IIIB	( +)	Endoscopy	( +)
3	68	Male	Operation of the anal fistula	(+), 40	No date	None	IIB	(-)	Local anesthesia	( +)
4	52	Female	Operation of the anal fistula	(+), 17	No date	None	IIIB	( +)	Fistulectomy	( +)
5	74	Male	Examination of the anal fistula	(−)	No date	None	IIA	(-)	Fistulectomy	No examination
6	74	Male	Examination of the anal fistula	(−)	11	HCRT	IIB	( +)	Local anesthesia	( +)
7	65	Male	Examination of the anal fistula	(−)	20	None	IIB	(-)	Endoscopy	( +)

*HCRT* hyperthermo-chemoradiotherapy, *PET* positron emission tomography

### Surgical results and pathological diagnosis

The surgical results of seven cases are shown in Table 2. Abdominoperineal amputation was performed in all cases and laparoscopic procedure was chosen in one case. The median surgery time was 422 min, median blood loss was 591 ml, and the median postoperative hospital stay was 25 days. Postoperative complications were found in five patients among whom, Clavien–Dindo classification of IIIa or higher was found in two patients.

**Table 2:** Surgical results of seven patients with cancer associated with anal fistula

Case	Laparoscopic surgery	Operation time (min)	Blood loss (ml)	Postoperative length of stay (day)	Complications	Clavien–Dindo classification	Residual tumor classification	Histological type	pT	pN	M	pStage
1	(−)	163	400	13	SSI	II	R0	muc	2	0	0	ypIIA
2	(−)	422	560	95	Pelvic abscess	IIIa	R0	muc	3	0	0	ypIIB
					Ileus							
3	(−)	520	1990	46	Discitis	II	R0	SCC	3	0	0	pIIB
4	(−)	467	1847	25	None	None	R0	muc	4	0	1	pIV
5	(−)	225	662	19	Neurogenic bladder	Iid	R1	muc	2	0	0	pIIA
6	(−)	294	591	99	SSI, pelvic abscess	IIIa	R0	tub1	3	0	0	ypIIB
7	(+)	446	239	13	None	None	R0	muc	3	0	0	pIIB

*SSI* surgical site infection, *SCC* squamous cell carcinoma

The postoperative pathological diagnosis revealed that the histological types were mucinous adenocarcinoma in five cases, tubular adenocarcinoma in one case, and squamous cell carcinoma in one case. The depth of invasion was pT2 in two cases, pT3 in four cases, and pT4 in one case. No lymph node metastasis was observed in any case. One case had isolated metastases in the ovary and was pathologically diagnosed with ovarian metastases. Pathological stage was IIA in two cases, IIB in four, and IV in 1. Downstaging was observed in two of three cases who received preoperative HCRT. Six patients underwent complete pathological resection and one patient who did not receive preoperative HCRT had a positive resection margin.

### Prognosis

Table 3 shows the prognosis after operation. In our department, postoperative adjuvant therapy is usually decided by a team consulting with the patient and considering the pathological results and the patient's general condition. Adjuvant therapy with radiotherapy and UFT/UZEL oral treatment was performed only for the patient with positive resection margin. The other six patients were followed up without any adjuvant therapy. Postoperative recurrence was observed in four cases (observation period was 237–1825 days, median: 875 days). The sites of recurrence were lungs, liver, pelvic lymph nodes, and inguinal lymph nodes. The case with recurrence of liver and inguinal lymph nodes underwent radical resection and has shown no further recurrences to date. For the patient with recurrent pelvic lymph nodes, heavy ion radiotherapy was performed, but multiple lung and subcutaneous metastases occurred 2 months after the end of therapy. Systemic chemotherapy is currently being performed for this patient. To date, one patient had recurrence in the lungs and died of cancer despite receiving the best supportive care.

**Table 3:** Prognosis of seven patients with cancer associated with anal fistula

Case	Postoperative therapy	Recurrence-free survival (day)	Recurrent site	Outcome	Overall survival (day)
1	None	175	Lung	Cancer death	237
2	None	569	Liver	Alive after recurrence resection	1269
3	None	875	None	Alive	875
4	None	330	Pelvic lymph node	Alive with recurrence	520
5	Radiation + UFT/UZEL	1825	None	Alive	1825
6	None	1825	None	Alive	1825
7	None	366	Inguinal lymph node	Alive after recurrence resection	477

## Discussion

Anorectal fistula cancer is defined as a developing tumor from the anal sinus or fistula as per the WHO classification [11]. The anorectal fistula cancer is reported to be associated with chronic inflammation that persists for more than 10 years, accounting for about 0.1% of anorectal fistula [12]. It is clinically difficult to make a diagnosis for the disease. Okada et al. reported a case in which preoperative pathological diagnosis was not made despite multiple biopsies and the diagnosis of malignant tumor was finally obtained from the resected specimen [1]. Consequently, in many cases, the diagnosis is made at an advanced stage [2]. It was reported that there were cases that died of multiple metastases early after surgery, highlighting the severity of the disease [13]. However, the pathophysiology and clinical features of anorectal fistula cancer are still unclear.

Rectal amputation is the common surgical procedure for anorectal fistula cancer, and combined resection, such as total pelvic exenteration, may also be performed according to the size of the tumor and the invading organs. The significance of inguinal lymph node dissection for anorectal fistula cancer is controversial. In this series of cases, inguinal lymph node recurrence was found in one case, suggesting the necessity to perform inguinal lymph node dissection according to the condition. The effectiveness of preoperative chemoradiotherapy has recently been reported [14]. In our case series, preoperative HCRT was performed for three cases, and R0 resection was achieved for all of them. It is difficult to secure surgical margin in surgery for anorectal fistula cancer because the extent of tumor is often unclear. According to reports, in the case of Crohn's disease, it has been reported that chemoradiotherapy increases adverse events, but in recent years it has been reported that irradiation can be performed with low toxicity due to advances in irradiation technology [15, 16]. Aggressive preoperative therapy including HCRT might be useful to obtain favorable surgical results.

**Fig. 1:**
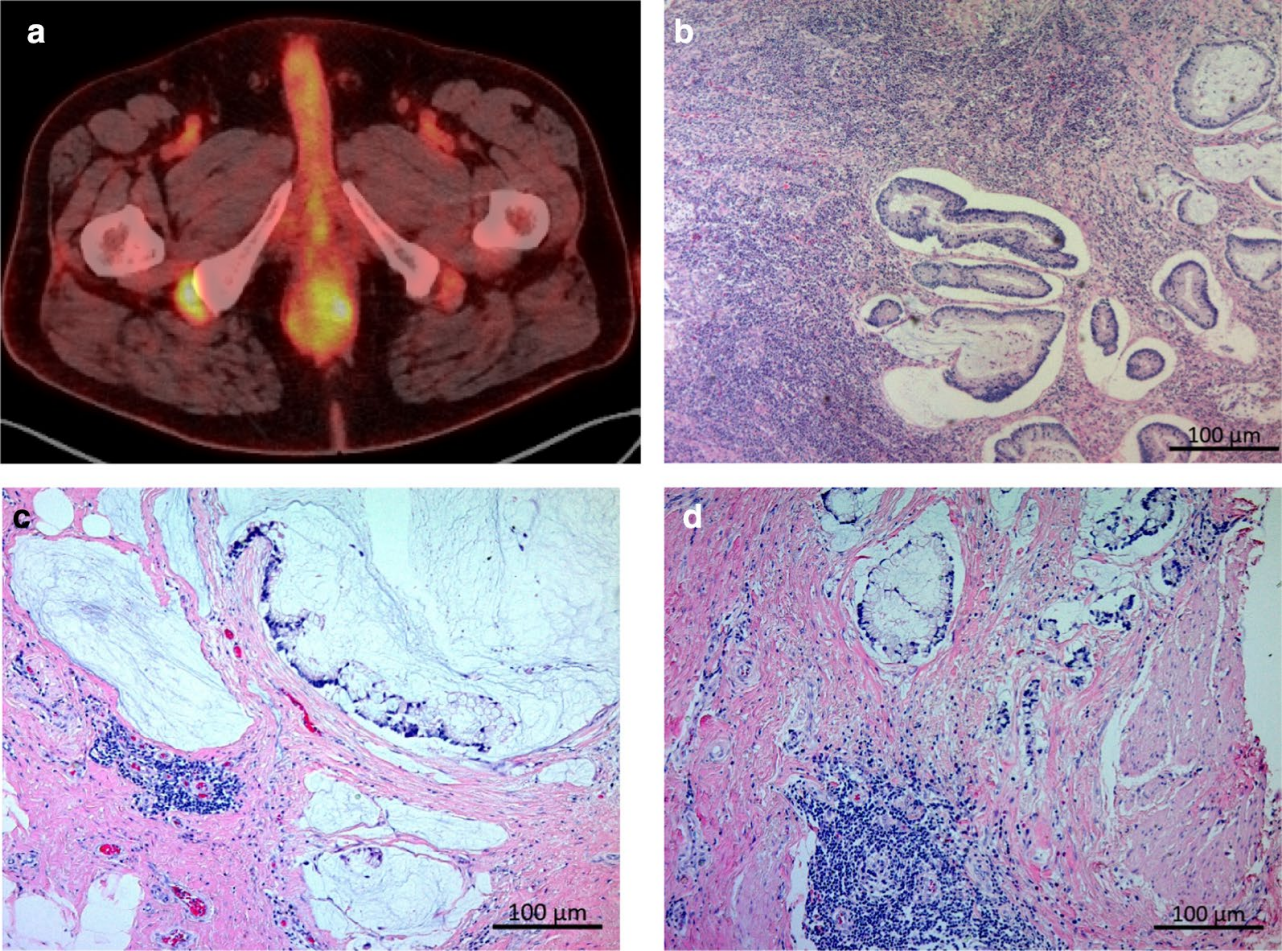
The findings of [^18^F]-fluoro-2-deoxy-d-glucose (FDG)-positron emission tomography/computed tomography (PET/CT) (**a**) and Hematoxylin–eosin staining of resected specimen (**b**, **c**, **d**) in the presented case of mucinous adenocarcinoma (case 7). **a** PET/CT image shows abnormal accumulation of FDG in the tumor. **b** Tumor cells are found floating in the abscess derived from the anorectal fistula. **c** Rich mucinous production is found in the tumor. Infiltration of inflammatory cells was also observed around the tumor. **d** The infiltration of many inflammatory cells was found around the tumor cells

The major histologic type of anorectal fistula cancer has been reported to be mucinous cancer [17]. Our study also found that mucinous cancer was the most frequent type of histology. Whiteford et al. reported that the sensitivity of FDG-PET/CT was low in mucinous adenocarcinoma of the colon and rectum [18]. However, in this study, all six patients with mucinous adenocarcinoma who received FDG-PET/CT demonstrated abnormal FDG accumulation (representative case is shown in Fig. 1). Since anorectal fistula cancer occurs at the inflammatory site and invades further, it is often accompanied by abscesses, fibrosis, and infiltration of inflammatory cells around the tumor [19]. It can be speculated that FDG is more likely to accumulate in anorectal fistula cancer, unlike normal mucinous cancer, because the tumor contains sites of inflammation [20].

Kodama et al. report that anorectal fistula cancer associated with Crohn's disease has a worse prognosis than usual anorectal fistula cancer [17]. It has been reported that the expression of epithelial-to-mesenchymal transition-related protein is enhanced in the anorectal fistula cancer in Crohn's disease, thus increasing the likelihood for metastasis [21]. In this study as well, recurrence was observed in three out of four cases with Crohn's disease (recurrence pattern: lung, liver, and pelvic lymph node). The number of patients with Crohn's disease is increasing [22], and consequently, it can be estimated that the number of patients with anorectal fistula cancer associated with Crohn's disease will also increase in the future.

The limitation of this study is that it is retrospective in nature and based on a small sample size as the cancer associated with anorectal fistula is very rare; thus, it may bias the results of our study. However, the design of large-scale studies to clarify the clinical features may be difficult or impractical due to the rarity of the disease. Much of what is known regarding the characteristics of the disease has been obtained through the accumulation of evidence from small-scale clinical cohorts. We believe the present study provides valuable insight into the perspectives of the treatment strategy.

## Conclusion

Anorectal fistula cancer is rare and difficult to be diagnosed. Mucinous adenocarcinoma associated with anorectal fistula tends to exhibit abnormal FDG accumulation by FDG-PET/CT. Preoperative HCRT may be effective to obtain pathological complete resection. Further accumulation of reported cases and improvement of treatment strategies are required.

## Abbreviations

FDG: [18F]-fluoro-2-deoxy-d-glucose
PET/CT: Positron emission tomography/computed tomography
HCRT: Hyperthermo-chemoradiotherapy
